# The relationship between self-compassion and self-worth in patients with traumatic brain injury: a latent profile analysis and examination of influencing factors

**DOI:** 10.3389/fpubh.2026.1730857

**Published:** 2026-02-12

**Authors:** Huijuan Zhang, Lili Zhao, Ling Li

**Affiliations:** Zhangjiakou First Hospital, Zhangjiakou, Hebei, China

**Keywords:** death anxiety, self-compassion, self-worth, social support, traumatic brain injury patients

## Abstract

**Background:**

Traumatic brain injury (TBI) often leads to post-traumatic syndrome, including symptoms such as dizziness, headaches, and tinnitus, significantly compromising patients’ quality of life. Self-compassion and self-worth, as key self-perception factors, have been widely demonstrated in previous research to buffer against negative self-evaluations, enhance self-worth, and improve quality of life. However, the internal heterogeneity of self-compassion and self-worth within the TBI patient population has not been sufficiently explored. This study aimed to identify potential subtypes based on the relationship between self-compassion and self-worth in TBI patients using latent profile analysis and to explore their influencing factors, thereby revealing individual differences and providing a basis for targeted interventions.

**Method:**

This cross-sectional study employed a convenience sampling method to recruit 586 diagnosed TBI patients between January and May 2025. Participants completed scales assessing self-compassion, self-worth, social support, and death anxiety to evaluate core variables. Latent profile analysis was conducted based on scores from the Self-compassion Scale and the Self-worth Scale to identify distinct profiles. Multinomial logistic regression analysis was used to examine the effects of demographic variables, injury-related factors, and psychological factors on profile membership.

**Result:**

This study found a significant positive correlation between self-compassion and self-worth in TBI patients. Latent profile analysis identified three distinct profiles: a ‘High self-compassion-high self-worth’ group, a ‘Moderate self-compassion-moderate self-worth’ group, and a ‘Moderate self-compassion-low self-worth’ group. Multinomial logistic regression analysis revealed that social support, death anxiety, and gender were significant factors influencing profile membership.

**Conclusion:**

This study uncovered heterogeneous profiles characterizing the relationship between self-compassion and self-worth in patients with traumatic brain injury and identified gender, death anxiety, and social support as key influencing factors. These findings underscore the necessity for targeted psychological interventions, such as enhancing social support and reducing death anxiety, to improve self-compassion in TBI patients, thereby fostering self-worth and quality of life.

## 1 Introduction

Traumatic brain injury (TBI) refers to damage to the skull, meninges, intracranial vessels, and brain tissue resulting from external mechanical forces (1, 2). Such injuries often induce acute neurological dysfunction, with long-lasting cognitive, emotional, and physical impairments (3, 4). Globally, TBI affects a substantial number of individuals, with disproportionately higher prevalence in low- and middle-income countries, mainly due to traffic accidents, falls, and violence (5, 6). Consequently, research has traditionally emphasized physiological rehabilitation and cognitive training (7, 8). For example, Martínez-Molina, Siponkoski (9) demonstrated that music interventions significantly improved executive functioning among individuals with TBI. In recent years, however, increasing attention has been given to addressing negative psychological factors to further enhance quality of life (8, 10), including symptoms such as anxiety, depression, and fear (11, 12). Nevertheless, insufficient attention has been given to the role of positive psychological resources in recovery—particularly the interaction between self-compassion and self-worth.

Self-compassion refers to a mindful, kind, and non-judgmental stance toward one’s own suffering. It encompasses three central elements: self-kindness, common humanity, and mindfulness (13, 14). In the context of TBI, self-compassion may act as a protective mechanism, supporting patients in coping with identity crises, functional impairment, and social isolation (15, 16). Individuals with higher self-compassion are more likely to approach personal limitations with tolerance, thus reducing anxiety and depression (17). However, due to executive dysfunction and challenges in emotion regulation, many individuals with TBI struggle to develop or sustain self-compassion (18). For example, prefrontal cortex injury may diminish the capacity for self-reflection, amplifying self-criticism and emotional numbness. Recent findings further indicate no significant differences in self-compassion or self-efficacy levels between patients with TBI and healthy peers (19, 20). Yet, the relationship between self-compassion and self-worth in individuals with TBI remains largely unexplored.

Self-worth, derived from conditional self-worth theory, reflects individuals’ sense of personal value based on achievements, social recognition, or intrinsic attributes (21, 22). Serving as a central pillar of mental health, self-worth is particularly sensitive during TBI rehabilitation (23). Traumatic events often destabilize patients’ physical functioning, social roles, and occupational identities, leading to sharp fluctuations in perceived self-worth (24). According to psychosocial development theory (25), self-worth is formed through an interaction between competence perception and social reinforcement. However, patients with TBI frequently suffer diminished self-worth due to functional deficits and societal stigma (26). Research has shown that low self-worth is significantly associated with depression, loneliness, and reduced treatment adherence among TBI survivors (27, 28). Strikingly, little is known about whether self-compassion functions as a preceding factor that bolsters self-worth. Ignoring this link is unfortunate, since higher self-compassion may help patients preserve self-worth through acceptance and self-acknowledgment, while lower self-compassion could exacerbate self-denial and self-criticism (29, 30).

Existing research often employs variable-centered analyses that presume psychological homogeneity within samples. In broader populations and chronic illness contexts, many studies consistently report a positive association between self-compassion and self-worth (31, 32). For instance, self-compassion has been found to enhance self-worth in women with chronic pain (33). Yet, some studies suggest weaker or domain-specific links, such as between self-compassion and appearance-contingent self-worth (34). In depressed women, self-compassion indirectly enhances self-worth through mechanisms such as self-acceptance and psychological capital (35). These inconsistencies may stem from not accounting for subgroup heterogeneity. For individuals with TBI, physical impairments and role disruption often interact with negative affect and social feedback, threatening both self-worth and self-compassion (26, 36). Conversely, heightened self-compassion promotes gentle self-care, perspective-taking on shared human suffering, and mindful nonjudgment—which collectively suppress self-denigration and catastrophizing, leading to stabilized self-worth, stronger adherence to rehabilitation, and improved social functioning (37, 38). Given the clinical diversity of TBI in injury severity, treatment burden, financial strain, and stigma, person-centered analytic approaches such as latent profile analysis (LPA) may uncover more nuanced profiles of self-compassion and self-worth compared with variable-centered models.

From the stress-buffering model of social support, social networks provide emotional, informational, and instrumental support, shaping both self-perception and emotion regulation (39, 40). For patients with TBI, however, trauma often disrupts social networks and strips away role functions (41), amplifying isolation and crises of self-worth. High levels of social support validate individuals’ limitations and reduce self-criticism, fostering greater self-kindness (42, 43). At the same time, social support helps integrate traumatic experiences into life narratives, rebuild positive identity, and reinforce self-worth (44). Thus, social support is likely to be a key contextual factor influencing profiles of self-compassion and self-worth.

A further crucial factor is death anxiety. Rooted in terror management theory, death anxiety emerges from awareness of mortality, which individuals buffer through bolstering cultural worldviews and self-worth (45). For patients with TBI—who often face sudden bodily vulnerability, functional limitations, and uncertainty regarding recovery—death-related concerns may become salient and may undermine adaptive self-related processes (46, 47). Specifically, heightened death-related thoughts have been shown to predict greater depressive symptoms among individuals with low self-worth, suggesting that mortality concerns can erode self-evaluations when self-worth resources are insufficient (48, 49). Conceptually, elevated death anxiety may also promote threat-focused (50), self-critical coping styles that are inconsistent with self-compassion, which relies on mindful and tolerant acceptance of human limitations (51, 52). Simultaneously, disruption of self-identity and social roles is frequently reported after TBI and is a recognized challenge to psychosocial functioning, potentially contributing to declines in self-worth (53, 54).

To address these research gaps, the present study applies LPA to identify overlooked psychosocial subgroups among patients with TBI. LPA is a person-centered, model-based clustering technique that identifies latent subgroups within a population based on individuals’ response patterns on continuous indicators (55, 56). Unlike variable-centered approaches that estimate average associations assuming population homogeneity (57, 58), LPA characterizes heterogeneity by estimating profile-specific means and assigning individuals to the most likely profile with quantifiable classification accuracy (e.g., entropy). LPA has been widely employed in clinical research, such as classifying pancreatic cancer patients based on psychological capital and death anxiety (59). Though previously used in TBI to explore heterogeneity in cognition, emotional regulation, and rehabilitation outcomes, LPA has not yet been applied to reveal combined distributions of self-compassion and self-worth.

The primary objective of this study is to identify distinct latent profiles characterizing the relationship between self-compassion and self-worth among TBI patients and to examine the demographic, clinical, and psychosocial factors associated with profile membership. A secondary objective is to provide empirically grounded recommendations for profile-specific psychological interventions.

Based on the theoretical frameworks outlined above, we propose the following hypotheses:

Hypothesis 1: Multiple distinct latent profiles of self-compassion and self-worth exist among TBI patients, reflecting heterogeneous patterns of self-perception. Specifically, we anticipate identifying at least three profiles representing varying levels of self-compassion and self-worth concordance.

Hypothesis 2: Social support serves as a protective factor for profile membership. Patients with higher perceived social support are more likely to belong to profiles characterized by elevated self-compassion and self-worth, whereas those with lower social support are more likely to belong to profiles marked by diminished self-worth.

Hypothesis 3: Death anxiety functions as a risk factor for profile membership. Patients with higher death anxiety are less likely to belong to profiles characterized by high self-compassion and self-worth, given that mortality salience may undermine self-compassionate coping and destabilize self-worth.

This study addresses three critical research questions aligned with these hypotheses:

1. Do distinct profiles of self-compassion and self-worth exist among TBI patients?
2. How are these profiles associated with demographic, clinical, and psychosocial characteristics?
3. How can such profiles inform individualized intervention strategies?

Through the application of LPA, this study aims to refine the psychological classification of TBI populations and provide empirical evidence to support tailored, profile-specific rehabilitation approaches. By linking profile characteristics to specific intervention modalities, we seek to bridge the gap between population-level psychological research and individualized clinical practice.

## 2 Methods

### 2.1 Participants

#### 2.1.1 Ethical considerations

This study strictly adhered to the principles of the Declaration of Helsinki and its subsequent amendments, and it was approved by the Institutional Review Board of the institution (Approval No.: 2025-LW-31). All participants were provided with detailed information prior to recruitment, including the study objectives, procedures, potential risks and benefits, the voluntary nature of participation, and the right to withdraw at any time. Written informed consent was obtained before inclusion. All data were anonymized and accessible only to the research team.

#### 2.1.2 Study design

A cross-sectional design was employed to examine the heterogeneity of the relationship between self-compassion and self-worth among TBI patients, as well as associated influencing factors. A cross-sectional design enabled data collection at a single time point to explore associations, rather than causal relationships. Participants were recruited using convenience sampling. Core variables assessed included self-compassion, self-worth, social support, and death anxiety.

In the present study, we focused on death anxiety as a theoretically and clinically salient form of existential threat that is directly implicated in self-worth regulation (terror management theory). While other anxiety domains (e.g., generalized anxiety, panic, post-traumatic stress–related anxiety) are also relevant after TBI, our aim was to examine whether an existentially grounded anxiety construct helps explain heterogeneity in the joint distribution of self-compassion and self-worth. Moreover, to minimize participant burden and avoid symptom exacerbation, we excluded individuals with current severe anxiety or depressive disorders; thus, the study was not designed to model clinical anxiety syndromes but rather to test the role of death anxiety as a profile-related correlate.

#### 2.1.3 Recruitment procedure

Data were collected between January and May 2025 in the neurosurgery outpatient and inpatient departments of four tertiary Class-A hospitals in Hebei Province, China. First, potential participants were screened through electronic medical records according to inclusion criteria. Next, the study was introduced both orally and via posters. Interested patients underwent eligibility interviews. Those deemed eligible provided informed consent and proceeded to complete the questionnaires, which were available in both paper-based and electronic formats (via QR code) to accommodate participant preference.

#### 2.1.4 Inclusion and exclusion criteria

Inclusion criteria: Age ≥18 years; Clinically confirmed TBI by neuroimaging with a disease course ≥3 months (to ensure stabilization of acute symptoms); Clear consciousness, adequate comprehension, and the ability to complete questionnaires; No severe communication barriers and capable of providing informed consent. Consciousness and comprehension were assessed using the Glasgow Coma Scale (GCS) (60), which evaluates eye response, verbal response, and motor response, with total scores ranging from 3 to 15. Higher scores indicate better consciousness levels. Patients with a GCS score ≤12 (moderate-to-severe coma) were excluded.

Exclusion criteria: Presence of other neurological disorders (e.g., Alzheimer’s disease, Parkinson’s disease) to minimize confounding; Current severe depressive or anxiety disorders, to prevent symptom exacerbation; Severe cognitive impairment or dysphasia; Refusal to participate or inability to complete the questionnaires. Cognitive function was assessed using the Mini-Mental State Examination (MMSE); patients scoring <24 were excluded (61).

Consciousness and comprehension were screened to ensure participants could provide informed consent and complete self-report questionnaires reliably. Although the Glasgow Coma Scale (GCS) is primarily used in acute care, we used the initial GCS score recorded at hospital admission (retrieved from electronic medical records) as a proxy for initial injury severity and level of consciousness, and excluded patients with GCS ≤ 12 (i.e., a history of moderate-to-severe coma), as such individuals are more likely to present with persistent disorders of consciousness and/or severe cognitive-communication limitations that may compromise questionnaire validity. To additionally verify current cognitive capacity at the time of survey administration (≥3 months post-injury), we applied the Mini-Mental State Examination (MMSE), excluding patients scoring < 24.

#### 2.1.5 Minimum sample size

Sample size estimation was based on statistical requirements for LPA and multivariate logistic regression. For LPA, referencing Nylund, Asparouhov (62), at least 300–500 participants are recommended to ensure model stability and clarity when assuming 3–5 potential profiles. For logistic regression, using G*power software with parameters set at *f*^2^ = 0.15, *α* = 0.05, 13 predictors, and 95% statistical power, the minimum required sample size was 189. Considering a 20% attrition rate, the minimum viable sample size was calculated as 227. Ultimately, this study recruited 586 participants—well exceeding the threshold and ensuring robust statistical power.

#### 2.1.6 Study sample

A total of 605 participants were recruited. Of these, 6 declined to provide informed consent, 3 were excluded due to other neurological disorders, and 10 were excluded because of incomplete questionnaires or response bias. The final effective sample comprised 586 participants, yielding an effective rate of 96.85%. Among them, 361 (61.60%) were men and 225 (38.40%) were women. Educational background was primarily middle to high school (*N* = 245, 41.80%), marital status was predominantly married (*N* = 336, 57.30%), and the majority resided in urban areas (*N* = 369, 63.00%). Most participants reported a monthly income of 3,001–5,000 (*N* = 190, 32.40%). The sample’s mean age was 35.14 ± 13.53 years. Detailed sociodemographic data are presented in Table 1.

**Table 1:** Demographic information of the participants.

Variables	Items	Number	Proportion
Gender	Female	225	38.40%
	Male	361	61.60%
Educational background	Primary school and below	162	27.60%
	Junior high school to Senior high school	245	41.80%
	Bachelor’s degree or above	179	30.50%
Marriage	Unmarried	158	27.00%
	Married	336	57.30%
	Divorced	73	12.50%
	Widowed	19	3.20%
Place of residence	Urban	369	63.00%
	Rural areas	217	37.00%
Monthly income	≤1,000	61	10.40%
	1,001–3,000	140	23.90%
	3,001–5,000	190	32.40%
	5,001–8,000	156	26.60%
	≥8,001	39	6.70%
Causes of trauma	Traffic accident	167	28.50%
	Tumble	130	22.20%
	Violent attack	121	20.60%
	Sport injury	168	28.70%
Trauma staging	Acute stage	132	22.50%
	Subacute stage	211	36.00%
	Chronic stage	243	41.50%

### 2.2 Measures tools

#### 2.2.1 Self-compassion scale

Self-compassion was assessed using the Short Form Self-compassion Scale developed by Raes, Pommier (63), consisting of 26 items across 6 dimensions: self-kindness (5 items), self-criticism (5 items), common humanity (4 items), isolation (4 items), mindfulness (4 items), and over-identification (4 items). The scale was adapted and validated for Chinese adolescents (Chinese version), demonstrating cultural applicability, reliability, and validity (64). It has also been widely applied in patient populations such as breast cancer (65), major depressive disorder (66), and brain injury (67, 68). Responses were rated on a 5-point Likert scale (1 = “almost never” to 5 = “almost always”). Positive subscales (self-kindness, common humanity, mindfulness) and negative subscales (self-criticism, isolation, over-identification) jointly generated an overall self-compassion score. Total scores range from 26 to 130, with higher scores indicating greater self-compassion. Confirmatory factor analysis (CFA) was performed in AMOS 29.0, and reliability testing in SPSS 27.0, showing good construct validity and internal consistency (χ^2^/df = 2.647, CFI = 0.904, GFI = 0.896, AGFI = 0.873, TLI = 0.891, RMSEA = 0.053; Cronbach’s *α* = 0.921).

#### 2.2.2 Self-worth scale

Self-worth was measured with the Contingencies of Self-worth Scale developed by Crocker, Luhtanen (69), consisting of 35 items across 7 domains: family support (5 items), competition (5 items), appearance (5 items), God’s love (5 items), academic competence (5 items), virtue (5 items), and others’ approval (5 items). The Chinese version was adapted by Cheng and Kwan (70) and later validated by Geng and Jiang (71). This scale has also been applied in populations such as cosmetic surgery patients (72), obsessive-compulsive disorder (73), and multiple sclerosis (74). Responses were rated on a 5-point Likert scale (1 = “strongly disagree” to 5 = “strongly agree”), with total scores ranging from 35 to 175. Higher scores indicate greater perceived self-worth. CFA and reliability testing confirmed strong psychometric properties (χ^2^/df = 2.632, CFI = 0.887, GFI = 0.845, AGFI = 0.825, TLI = 0.880, RMSEA = 0.053; Cronbach’s *α* = 0.950).

#### 2.2.3 Social support scale

Perceived social support was assessed using the Multidimensional Scale of Perceived Social Support (MSPSS) developed by Zimet, Dahlem (75). The MSPSS includes 12 items across three subscales: significant others (4 items), family (4 items), and friends (4 items). The Chinese version was validated in university populations (76) and has demonstrated strong reliability in brain injury populations (77, 78). Responses were rated on a 5-point Likert scale (1 = “strongly disagree” to 5 = “strongly agree”). A mean score across all items was calculated, with possible scores ranging from 1 to 5; higher scores reflect stronger perceived social support. In the subsequent multinomial logistic regression analysis, Odds Ratios for social support represent the change in odds associated with each 1-point increase on this 5-point scale. Good model fit and reliability were confirmed (χ^2^/df = 2.416, CFI = 0.962, GFI = 0.964, AGFI = 0.947, TLI = 0.953, RMSEA = 0.049; Cronbach’s *α* = 0.870).

#### 2.2.4 Death anxiety scale

Death anxiety was measured by the Death Anxiety Scale (DAS) originally developed by Templer (79). It consists of 15 items assessing anxiety responses to death, illness, surgery, time, war, and exposure to corpses. The Chinese version was adapted and validated in colorectal cancer patients (80). The scale has been widely applied in cancer (81), chronic illness (82), and chronic back pain (83). Participants rated items on a 5-point Likert scale (1 = “strongly disagree” to 5 = “strongly agree”). A mean score across all items was calculated, with possible scores ranging from 1 to 5; higher scores indicate greater death anxiety. In the subsequent multinomial logistic regression analysis, Odds Ratios for death anxiety represent the change in odds associated with each 1-point increase on this 5-point scale. CFA and reliability analyses supported adequate construct validity and internal consistency (χ^2^/df = 2.279, CFI = 0.937, GFI = 0.954, AGFI = 0.939, TLI = 0.926, RMSEA = 0.047; Cronbach’s *α* = 0.822).

### 2.3 Statistical analyses

All statistical analyses were conducted using SPSS 27.0, AMOS 29.0, and Mplus 8. First, CFA was performed in AMOS 29.0 to evaluate model fit for the core measures. Second, SPSS 27.0 was employed for common method bias testing, descriptive statistics, and correlation analyses. Third, Mplus 8 was used to construct a five-profile latent profile model of self-compassion and self-worth; selection of the optimal model was based on Akaike Information Criterion (AIC), Bayesian Information Criterion (BIC), Adjusted BIC (aBIC), and Entropy. Subgroup profiles for the optimal model were then generated. In LPA, individuals are probabilistically assigned to the latent profile with the highest posterior probability; entropy was used to evaluate classification precision. Fourth, univariate tests assessed differences in demographics, social support, and death anxiety across self-compassion - self-worth latent profiles. Finally, multivariate logistic regression tested the predictive value of significant variables from univariate analyses.

Model fit indices included: Comparative Fit Index (CFI ≥ 0.80 acceptable), Goodness-of-Fit Index (GFI ≥ 0.80), Adjusted Goodness-of-Fit Index (AGFI ≥ 0.80), Tucker-Lewis Index (TLI ≥ 0.80), and Root Mean Square Error of Approximation (RMSEA ≤ 0.08). Profile fit criteria included smaller values of AIC, BIC, and aBIC, while Entropy > 0.80 indicated excellent classification quality. A *p*-value < 0.05 was considered statistically significant.

## 3 Result

### 3.1 Common method bias analysis

To further reduce common method bias, this study employed anonymization and confidentiality during the data collection phase to mitigate participants’ social desirability tendencies and evaluation concerns. Subsequently, we utilized Harman’s single-factor test, conducting exploratory factor analysis on all variable measurement items to examine the unrotated factor solution. The results indicated that the first factor explained 23.94% of the variance, which did not exceed the threshold of 40%, suggesting that this study does not suffer from serious common method bias.

### 3.2 Descriptive statistics and correlation analysis

We conducted descriptive statistics and correlation analysis on demographic information, self-compassion, self-worth, social support, and death anxiety, as presented in Tables 2, 3. The levels of self-compassion (*M* = 3.142, SD = 0.657), self-worth (*M* = 3.121, SD = 0.685), social support (*M* = 3.015, SD = 0.736), and death anxiety (*M* = 3.293, SD = 0.679) were all above the midpoint (*M* = 2.5). Meanwhile, the skewness of the core variables ranged from −0.787 to 0.061, and kurtosis ranged from −0.193 to −0.458. According to Kline (84) findings, the core variables in this study exhibited an approximately normal distribution.

**Table 2:** Descriptive statistical analysis of core variables.

Variables	*M*	SD	Skewness	Kurtosis
Self-compassion	3.142	0.657	0.061	−0.127
Self-worth	3.121	0.685	−0.204	−0.193
Social support	3.015	0.736	−0.163	−0.100
Death anxiety	3.293	0.679	−0.787	0.458

**Table 3:** Correlation analysis of demographic information and core variables.

Variable	1	2	3	4	5	6	7	8	9	10	11	12
Self-compassion	1											
Self-worth	0.571**	1										
3.Social support	0.306**	0.622**	1									
4.Death anxiety	−0.480**	−0.526**	−0.144**	1								
5.Gender	−0.242**	−0.367**	−0.146**	0.265**	1							
Age	−0.002	0.008	0.002	0.012	−0.012	1						
Education background	−0.116**	−0.185**	−0.144**	0.102*	0.016	−0.406**	1					
Marriage	0.044	0.058	0.046	−0.057	0.014	0.680**	−0.263**	1				
Place of residence	0.067	−0.003	−0.062	−0.044	0.024	0.001	−0.001	0.110**	1			
Monthly income	0.039	0.03	0.001	0.028	0.004	−0.122**	−0.076	−0.014	0.011	1		
Causes of trauma	0.005	−0.100*	−0.098*	0.04	0.06	−0.102*	0.100*	−0.100*	−0.094*	−0.033	1	
Trauma staging	−0.013	0.02	0.075	0.02	−0.02	−0.029	−0.018	−0.031	−0.005	0.015	−0.046	1

*p < 0.05; **p < 0.01.

Table 3 displays the complete correlation matrix. The study found that self-compassion had a significant positive correlation with self-worth (*r* = 0.571, *p* < 0.01), a significant positive correlation with social support (*r* = 0.306, *p* < 0.01), and a significant negative correlation with death anxiety (*r* = −0.408, *p* < 0.01). Self-worth also showed a significant positive correlation with social support (*r* = 0.626, *p* < 0.01) and a significant negative correlation with death anxiety (*r* = −0.526, *p* < 0.01). Social support was significantly negatively correlated with death anxiety (*r* = −0.144, *p* < 0.01).

Regarding demographic information, education and gender significantly correlated with self-compassion, self-worth, social support, and death anxiety (*p* < 0.01). The cause of trauma significantly correlated with self-worth and social support (*p* < 0.05). However, age, marital status, residence, monthly income, and trauma phase showed no significant correlations with the core variables (*p* > 0.05).

### 3.3 Latent profile analysis of self-compassion and self-worth

Using the dimension scores of self-compassion and self-worth as indicators, we employed Mplus 8 software to construct a latent profile model for TBI patients, categorizing them into 1 to 5 classes. As shown in Table 4, with an increasing number of latent profiles, the values of AIC, BIC, and aBIC consistently decreased, indicating an improvement in the fit of the latent profile model. Compared to the two-class latent profile model (Entropy = 0.934), the three-class latent profile model exhibited superior classification clarity, providing more reliable results (Entropy = 0.946). To determine whether adding an additional profile significantly improved model fit, we used the Lo–Mendell–Rubin adjusted likelihood ratio test (LMR-LRT), which compares a k-profile model against a (k–1)-profile model. The LMR-LRT was significant for the three-profile model (*p* = 0.016; 3 vs. 2 profiles), suggesting that the three-profile solution fit significantly better than the two-profile solution. In contrast, the LMR-LRT was not significant for the four-profile model (*p* = 0.172; 4 vs. 3 profiles), indicating that adding a fourth profile did not provide a statistically significant improvement over the three-profile solution. Considering the nonsignificant incremental gain for the 4-profile model, the higher entropy for the three-profile solution (0.946), and the principle of parsimony and interpretability, we selected the three-profile model as the optimal solution.

**Table 4:** One to five fitting indicators of potential profile models.

Profile	AIC	BIC	aBIC	Entropy	LMR(P)	BLRT(P)	Smallest proportion per class
1	18299.824	18413.530	18330.990				
2	15535.798	15710.731	15583.745	0.934	<0.001	<0.001	0.653/0.347
3	14368.002	14604.162	14432.731	0.946	0.016	<0.001	0.188/0.258/0.554
4	13485.930	13783.316	13567.440	0.936	0.172	<0.001	0.231/0.467/0.066/0.236
5	13140.906	13499.518	13239.197	0.914	0.139	<0.001	0.136/0.407/0.066/0.224/0.167

To evaluate the robustness of the three-class solution, particularly given that Class 1 comprised 18.8% of the sample, we conducted several sensitivity analyses. While this proportion approaches commonly discussed thresholds for small class sizes in LPA, it substantially exceeds the recommended minimum criteria. Methodological guidelines suggest caution for classes containing fewer than 5% of the sample or fewer than 25 individuals (85, 86). Class 1, with 110 participants, far exceeds these thresholds. We performed split-sample cross-validation by randomly dividing the sample into two halves. Independent LPA on each subsample consistently yielded a three-class solution, with Class 1 proportions of 17.4 and 19.8% respectively, demonstrating stability across random subsamples.

**Figure 1:**
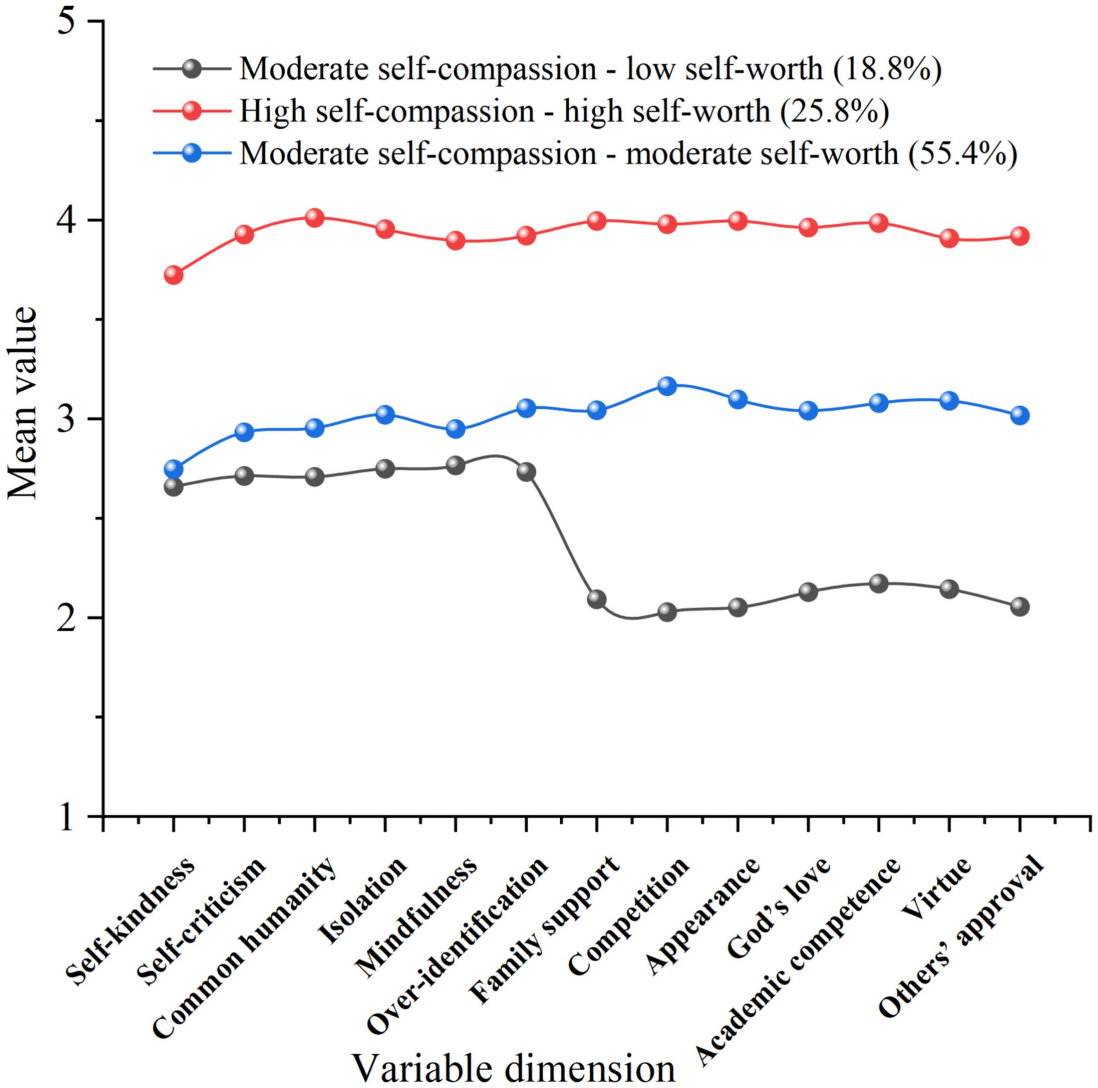
Latent profile plot of standardized mean scores (*z*-scores) across the measured dimensions of self-compassion and self-worth. The *x*-axis lists each dimension in the order used in the latent profile analysis: Self-kindness, Self-criticism, Common humanity, Isolation, Mindfulness, Over-identification, Family support, Competition, Appearance, God’s love, Academic competence, Virtue, Others’ approval. Higher values indicate higher levels on the corresponding dimension.

Based on the analysis results in Table 4, we utilized Origin 2021 software to plot the profile graph of the three-class latent profile model, as shown in Figure 1. Figure 1 illustrates that the first latent subgroup displays solid recognition and positive evaluation of self-compassion and self-worth, labeled as “High self-compassion – high self-worth,” comprising 25.8% of the participants. The second latent subgroup exhibits moderate recognition and evaluation of self-compassion and self-worth, named “Moderate self-compassion - moderate self-worth,” making up 55.4%. The third latent subgroup represents patients with moderate self-compassion and lower self-worth, identified as “Moderate self-compassion – low self-worth,” accounting for 18.8%.

### 3.4 Univariate analysis of latent subgroups

Before conducting univariate comparisons across subgroups, we first determined the optimal number of latent profiles. We fitted a series of LPA models with increasing numbers of profiles using self-compassion and self-worth indicators. Model fit improved from 2 to 3 profiles, as reflected by lower information criteria and a significant LMR-LRT for the 3-profile model (*p* = 0.016; 3 vs. 2 profiles). In contrast, the LMR-LRT for the 4-profile model was not significant (*p* = 0.172; 4 vs. 3 profiles), indicating that adding a fourth profile did not yield a statistically meaningful improvement over the 3-profile solution. The 3-profile solution also showed high classification accuracy (entropy = 0.946) and yielded interpretable profiles with adequate sample sizes. Therefore, we selected the 3-profile model for subsequent analyses.

After selecting the three-profile solution, we compared demographic, injury-related, and psychosocial variables across the latent classes using univariate tests (χ^2^ tests for categorical variables; ANOVA or nonparametric tests as appropriate for continuous variables). Variables showing significant between-class differences were then entered into a multinomial logistic regression model to identify independent correlates of class membership.

Significant gender differences were observed (*χ*^2^ = 75.138, *p* < 0.001), with a higher proportion of women in Class 1 (14.4%) and Class 3 (40.1%), while Class 2 had an even higher female proportion (73.0%), indicating that women are more likely to fall into the lower self-worth profile. Educational level differences were significant (*χ*^2^ = 21.774, *p* < 0.001), with a higher proportion of individuals in Class 2 holding a bachelor’s degree or higher (24.1%), while Classes 1 and 3 had a greater proportion of individuals with lower educational levels. There were no significant differences in marital status (*χ*^2^ = 7.258, *p* = 0.298) and monthly income (*χ*^2^ = 10.525, *p* = 0.230), suggesting these factors do not distinguish the profiles.

Differences in residence were marginally significant (*χ*^2^ = 6.471, *p* = 0.039), with a slightly higher proportion of urban residents in Class 3 (77.8%), while Classes 1 (61.9%) and 2 (66.2%) had relatively lower proportions. The cause of injury was significantly associated with profile membership (χ^2^ = 18.157, *p* = 0.006), with traffic accidents being more prevalent in Class 3 (36.9%), whereas sports injuries dominated Class 2 (30.8%). Differences in injury phases were significant (χ^2^ = 12.533, *p* = 0.014), with chronic phase patients over represented in Class 3 (51.1%) and acute phase patients more prevalent in Class 2 (24.1%).

There were no significant age differences among profiles (*F* = 0.184, *p* = 0.832). However, perceived social support showed significant differences (*F* = 173.645, *p* < 0.001), with Class 2 having the highest level (*M* = 3.527, SD = 0.789), followed by Class 3 (*M* = 3.061, SD = 0.736), and Class 1 the lowest (*M* = 2.164, SD = 0.549). A one-way ANOVA confirmed all pairwise comparisons were significant (*p* < 0.001). Death anxiety exhibited a similar pattern (*F* = 267.243, *p* < 0.001): Class 2 scored the lowest (*M* = 2.494, SD = 0.617), Class 1 was moderate (*M* = 3.522, SD = 0.446), and Class 3 was the highest (*M* = 3.584, SD = 0.439), with all pairwise comparisons being significant (*p* < 0.001).

These univariate results emphasize that the profile with higher self-compassion and self-worth (Class 2) is associated with greater social support, lower death anxiety, higher education levels, and specific injury characteristics, while the lower profile (Class 1) shows the opposite trend (Table 5).

**Table 5:** Univariate analysis of potential subgroups.

Variable	Items	Class 1	Class 2	Class 3	χ^2^/F	p
Gender	Female	14	97	114	75.138	<0.001
	Male	94	53	214		
Educational background	Primary school and below	24	57	81	21.774	<0.001
	Junior high school to Senior high school	36	61	148		
	Bachelor’s degree or above	48	32	99		
Marriage	Unmarried	27	31	100	7.258	0.298
	Married	64	90	182		
	Divorced	15	23	35		
	Widowed	2	6	11		
Place of residence	Urban	60	88	221	6.471	0.039
	Rural areas	48	62	107		
Monthly income	≤1,000	9	16	36	10.525	0.230
	1,001–3,000	35	32	73		
	3,001–5,000	39	47	104		
	5,001–8,000	19	42	95		
	≥8,001	6	13	20		
Causes of trauma	Traffic accident	18	44	105	18.157	0.006
	Tumble	25	42	63		
	Violent attack	23	23	75		
	Sport injury	42	41	85		
Trauma staging	Acute stage	36	32	64	12.533	0.014
	Subacute stage	29	63	119		
	Chronic stage	43	55	145		
Age		35.58 ± 12.81	34.61 ± 12.59	35.24 ± 14.19	0.184	0.832
Social support		2.164 ± 0.549	3.527 ± 0.789	3.061 ± 0.736	173.645	<0.001
Death anxiety		3.522 ± 0.446	2.494 ± 0.617	3.584 ± 0.439	267.243	<0.001

Class 1: Moderate self-compassion - low self-worth group; Class 2: High self-compassion - high self-worth group; Class 3: Moderate self-compassion - moderate self-worth group.

### 3.5 Multivariate logistic regression analysis of latent subgroups

To identify independent predictors of profile membership, we performed multivariate logistic regression using the significant variables from the univariate analysis (*p* < 0.05: gender, education, residence, cause of injury, injury phase, social support, death anxiety). Class 3 (“Moderate self-compassion - moderate self-worth”) was used as the reference category due to its maximum sample size and intermediate positioning. Two models were estimated, comparing Class 1 to Class 3 and Class 2 to Class 3, with results presented in Table 6. Notably, for continuous variables (social support and death anxiety), scores were calculated as mean item scores on 5-point Likert scales (range: 1–5), and the reported Odds Ratios represent the change in odds for each 1-point increase on these scales.

**Table 6:** Multiple regression analysis (with class 3 as the reference group).

Potential subgroups	Variables	Dimensionality	Regression Coefficient	Standard Error	Wald χ^2^	P	OR	LLCI	ULCI
Class 1	Social support		−3.009	0.337	79.628	<0.001	0.049	0.025	0.096
	Death anxiety		0.331	0.351	0.889	0.346	1.392	0.700	2.771
	Gender	Female	−1.3	0.392	11.03	0.001	0.272	0.126	0.587
		Male (refer)							
	Educational background	Primary school and below	−0.464	0.398	1.356	0.244	0.629	0.288	1.373
		Junior high school to Senior high school	−0.349	0.341	1.045	0.307	0.705	0.361	1.377
		Bachelor’s degree or above (refer)							
	Place of residence	Urban	−0.107	0.317	0.113	0.736	0.899	0.483	1.672
		Rural areas (refer)							
	Causes of trauma	Traffic accident	−0.649	0.408	2.532	0.112	0.522	0.235	1.162
		Tumble	−0.631	0.426	2.200	0.138	0.532	0.231	1.225
		Violent attack	−0.763	0.415	3.375	0.066	0.466	0.207	1.052
		Sport injury (refer)							
	Trauma staging	Acute stage	0.228	0.367	0.383	0.536	1.256	0.611	2.58
		Subacute stage	−0.452	0.373	1.466	0.226	0.636	0.306	1.323
		Chronic stage (refer)							
Class 2	Social support		1.914	0.303	39.872	<0.001	6.782	3.744	12.287
	Death anxiety		−3.938	0.395	99.555	<0.001	0.019	0.009	0.042
	Gender	Female	1.249	0.365	11.676	0.001	3.485	1.703	7.132
		Male (refer)							
	Educational background	Primary school and below	1.133	0.504	5.054	0.025	3.104	1.156	8.335
		Junior high school to Senior high school	0.616	0.466	1.745	0.187	1.851	0.742	4.614
		Bachelor’s degree or above (refer)							
	Place of residence	Urban	−0.342	0.377	0.825	0.364	0.71	0.34	1.486
		Rural areas (refer)							
	Causes of trauma	Traffic accident	−0.444	0.479	0.859	0.354	0.642	0.251	1.64
		Tumble	0.262	0.479	0.299	0.584	1.3	0.508	3.326
		Violent attack	−1.045	0.559	3.490	0.062	0.352	0.118	1.053
		Sport injury (refer)							
	Trauma staging	Acute stage	0.656	0.473	1.923	0.165	1.927	0.762	4.872
		Subacute stage	0.236	0.407	0.336	0.562	1.266	0.57	2.808
		Chronic stage (refer)							

Class 1: Moderate self-compassion - low self-worth group; Class 2: High self-compassion - high self-worth group.

For the comparison of Class 1 (“Moderate self-compassion - low self-worth”) to Class 3: social support emerged as a significant protective factor (*β* = −3.009, SE = 0.337, Wald χ^2^ = 79.628, *p* < 0.001, OR = 0.049, 95% CI = [0.025, 0.096]), indicating that higher social support significantly reduces the likelihood of falling into this low self-worth profile. This indicates that for every 1-point increase in social support on the 5-point scale, the odds of belonging to the low self-worth profile (Class 1) versus the moderate profile (Class 3) decrease by approximately 95% (OR = 0.049). Given that the sample mean for social support was 3.015 (SD = 0.736), a 1-point increase represents a substantial shift of approximately 1.4 standard deviations. Death anxiety was not significant (*β* = 0.331, SE = 0.351, Wald χ^2^ = 0.889, *p* = 0.346, OR = 1.392, 95% CI = [0.700, 2.771]). Gender was a key predictive factor, with women less likely to fall into Class 1 (*β* = −1.300, SE = 0.392, Wald χ^2^ = 11.030, *p* = 0.001, OR = 0.272, 95% CI = [0.126, 0.587]); specifically, women’s odds of falling into the low self-worth profile were approximately 73% lower than men’s. Education, residence, cause of injury, and injury phase did not reach significance (all *p* > 0.05), indicating that these effects were weakened when controlling for other variables.

For the comparison of Class 2 (“High self-compassion - high self-worth”) to Class 3: social support was a strong positive predictor (*β* = 1.914, SE = 0.303, Wald χ^2^ = 39.872, *p* < 0.001, OR = 6.782, 95% CI [3.744, 12.287]), with higher levels increasing the likelihood of falling into the high profile. This indicates that for every 1-point increase in social support on the 5-point scale, the odds of belonging to the high profile (Class 2) versus the moderate profile (Class 3) increase by a factor of approximately 6.8. Death anxiety served as a protective factor (*β* = −3.938, SE = 0.395, Wald χ^2^ = 99.555, *p* < 0.001, OR = 0.019, 95% CI = [0.009, 0.042]); for every 1-point increase in death anxiety, the odds of belonging to the high profile decrease by approximately 98% (OR = 0.019), underscoring the powerful negative association between existential anxiety and optimal self-perception profiles. Gender was significant, with women having approximately 3.5 times higher odds of belonging to Class 2 compared to men (*β* = 1.249, SE = 0.365, Wald *χ*^2^ = 11.676, *p* = 0.001, OR = 3.485, 95% CI = [1.703, 7.132]). Education showed a significant effect for the lowest category: individuals with primary school education or below had approximately 3.1 times higher odds of belonging to the high profile compared to those with bachelor’s degree or above (*β* = 1.133, SE = 0.504, Wald χ^2^ = 5.054, *p* = 0.025, OR = 3.104, 95% CI = [1.156, 8.335]), while the middle education category (junior high to senior high school) was not significant (*p* = 0.187). Residence, cause of injury, and injury phase were not significant (all *p* > 0.05).

Overall, these multivariate results underscore social support and death anxiety as robust psychosocial predictors of latent profile membership, with gender and education exerting additional independent effects. Demographic and injury-related factors appeared less critical when controlling for psychosocial variables, highlighting potential intervention targets in the rehabilitation of TBI patients.

## 4 Discussion

### 4.1 Latent profile analysis of self-compassion and self-worth

This study identified three heterogeneous subtypes of the relationship between self-compassion and self-worth in patients with TBI through LPA: the High self-compassion - high self-worth group (Class 2, 25.8%), the Moderate self-compassion - moderate self-worth group (Class 3, 55.4%), and the Moderate self-compassion - low self-worth group (Class 1, 18.8%). This finding reveals significant internal heterogeneity in self-perception within this patient population, contrasting with previous variable-centered research methods that often assume homogeneity and overlook individual differences. Specifically, patients in the High self-compassion - high self-worth group demonstrated elevated levels of both self-compassion and self-worth, potentially reflecting their stronger abilities for self-acceptance and positive self-evaluation mechanisms, thereby buffering against the negative impacts of trauma. In contrast, the Moderate self-compassion - moderate self-worth group, as the largest subtype, exhibited a balanced level of self-perception, indicating that most patients maintained relatively stable self-regulation strategies post-trauma. However, patients in the Moderate self-compassion – low self-worth group displayed moderate self-compassion but significantly lower self-worth, highlighting a potential disconnect where self-compassion did not effectively translate into self-worth for some individuals, possibly stemming from identity crises and loss of social roles triggered by trauma.

These profile patterns partially align with findings in existing literature on chronic illness and post-traumatic groups. For example, similar LPA studies in breast cancer patients have identified high, moderate, and low self-compassion subtypes (87), confirming that the high self-compassion group is associated with better psychological adjustment. However, this study uniquely incorporates both self-compassion and self-worth in the LPA indicators, extending the limitations of previous research that focused solely on single variables. The imbalanced pattern observed in the Moderate self-compassion - low self-worth group may arise from specific neurocognitive impairments associated with TBI, such as difficulties in reflection and emotional regulation due to frontal lobe dysfunction, which hinder the transformation of self-compassion into self-worth (88). This heterogeneity emphasizes the nonlinear characteristics of psychological recovery post-trauma: while self-compassion can serve as a protective factor, its effectiveness depends on individual backgrounds and trauma severity (16). Additionally, the proportion of low self-worth subtypes among TBI patients is relatively low, which may be attributed to the longer duration of illness in this sample (predominantly in the chronic phase), allowing some patients to reconstruct self-perception through the rehabilitation process.

From a theoretical perspective, these profiles support the conditional self-worth theory, wherein self-worth is influenced by achievements and social recognition. TBI often leads to functional loss and social isolation, resulting in fluctuations in self-worth (24). Meanwhile, self-compassion, as a buffering mechanism, may strengthen the stability of self-worth in the high-profile group through components of self-kindness and mindfulness (89). However, the disconnect in the moderate to low profile group suggests that simply enhancing self-compassion may be insufficient to restore self-worth, especially in the presence of death anxiety or insufficient social support. This finding provides insights for clinical practice: personalized interventions should be tailored to different profiles, such as emphasizing self-worth reconstruction training (e.g., cognitive behavioral therapy combined with self-compassion meditation) for the moderate to low group, while focusing on maintenance strategies for the high group to prevent potential declines. In summary, the results of the LPA in this study not only enrich the empirical foundation of psychological heterogeneity in TBI but also lay a theoretical framework for precise psychological interventions.

### 4.2 Analysis of influencing factors for latent subgroups

This study revealed key factors influencing the latent profile membership of self-compassion and self-worth in patients with TBI through multivariate logistic regression analysis, including social support, death anxiety, gender, and education level. Residence, cause of injury, and injury phase did not show independent predictive effects after controlling for other variables. This finding emphasizes the central role of psychosocial factors in the heterogeneity of self-perception post-trauma, resonating with social support buffering models and fear management theories. Specifically, social support emerged as a strong protective factor, positively correlating with the High self-compassion - high self-worth group and negatively correlating with the Moderate self-compassion - low self-worth group. This indicates that adequate social support can enhance patients’ self-kindness and stability of self-worth through emotional validation and resource provision (90). This result aligns with existing literature on TBI patients, where perceived social support significantly reduces psychological fatigue and feelings of isolation, thereby promoting positive self-perception reconstruction (91). Further research has shown that social support plays a crucial role in recovery from TBI, improving emotional regulation and buffering against self-criticism triggered by trauma (92, 93). However, the average level of social support in this study’s sample was relatively high, which may have amplified its protective effects; future studies should validate its threshold in low-support groups to optimize intervention strategies.

Death anxiety also served as another independent predictive factor, negatively correlating with the High self-compassion - high self-worth group, suggesting that lower death anxiety helps maintain a positive self-perception profile, while higher anxiety may disrupt the buffering mechanisms of self-compassion, leading to a decline in self-worth. This association supports the view of death anxiety as a cross-diagnostic construct, often amplifying negative self-evaluations in trauma patients by activating existential threats. In the context of TBI patients, death anxiety may arise from awareness of functional loss and life fragility, closely linked to low self-worth and depressive symptoms (94). Although death anxiety did not reach significant independent effects in the low-profile group, its overall negative correlation indicates it may influence profile membership through indirect pathways. The moderate level of death anxiety among TBI patients may reflect the abruptness of trauma rather than a gradual process, yet it still emphasizes the need to integrate existential therapies (e.g., meaning-oriented interventions) to reduce anxiety and enhance self-worth.

Gender differences were significantly evident in this study, with females more likely to belong to the High self-compassion - high self-worth group, while males were more prone to the low profile group. This may stem from women having higher baseline levels of self-compassion, including stronger components of self-kindness and mindfulness (95, 96). Existing literature confirms that women often report higher scores on self-compassion scales, although they may also exhibit higher scores on non-compassion dimensions (e.g., self-criticism) (95, 97). The gender differences in TBI patients may further amplify: women excel in emotional empathy and self-concept reconstruction, while men may suppress expressions of self-compassion due to social norms (e.g., rigid masculinity), leading to a greater vulnerability in self-worth. This finding aligns with gender differences in overall outcomes for TBI, where women face more challenges in physiological recovery but demonstrate stronger psychological adaptability.

Education level emerged as an additional predictive factor, with the low education group (elementary school or below) more likely to belong to the High self-compassion - high self-worth group. This counterintuitive result may indicate that individuals with lower education levels rely more on intrinsic sources of self-worth (e.g., familial support or personal resilience) rather than external achievements (e.g., occupational recognition), thereby buffering the impact of trauma on self-perception. Literature indicates that education level positively correlates with self-efficacy and quality of life in chronic disease patients, with higher educated individuals often demonstrating better self-care (98, 99). However, lower educated individuals might develop stronger internal compassion through simplified cognitive frameworks. In chronic illness populations, low education is associated with higher depression; yet, the association of high profiles in this study suggests that education may influence self-worth through health literacy. This finding challenges traditional assumptions and emphasizes the need to consider the interplay of education and cultural context: in low-income areas, lower educated patients may benefit more from community support. Other factors, such as residence, cause of injury, and injury phase, were not significant in the multivariate analysis, suggesting their effects may be mediated by psychosocial variables (e.g., social support absorbing urban–rural differences), which aligns with the limitations of variable-centered studies.

### 4.3 Education level interpretation

Education level emerged as an additional predictive factor with a seemingly paradoxical pattern: individuals with the lowest education level (primary school or below) demonstrated approximately 3.1 times higher odds of belonging to the High self-compassion - high self-worth group compared to those with bachelor’s degree or above. This counterintuitive finding warrants careful interpretation within the Chinese cultural context and consideration of multiple explanatory mechanisms.

First, this pattern may reflect the influence of collectivist cultural values predominant in Chinese society, particularly among older and less formally educated populations. In collectivist cultures, self-worth is often more strongly anchored in relational and communal domains rather than individual achievements (100, 101). For individuals with lower formal education, self-worth may be primarily derived from family roles, community standing, and moral character rather than educational or occupational accomplishments. Consequently, the functional impairments and occupational disruptions caused by TBI may pose less threat to their self-worth foundations compared to highly educated individuals whose self-worth is more contingent upon achievement and professional identity.

Second, traditional Chinese philosophical frameworks, particularly Confucian concepts of self-cultivation and Buddhist teachings on acceptance and non-attachment, may provide cognitive resources that promote self-compassion independent of educational attainment (102). These philosophical traditions, often more deeply embedded in rural and less-educated communities through oral transmission and cultural practices, emphasize inner contentment, acceptance of life circumstances, and compassionate self-treatment during hardship. Such cultural resources may enable individuals with lower formal education to maintain self-compassion and self-worth despite traumatic experiences.

Third, structural differences in social support networks may partially explain this finding. In Chinese society, individuals with lower education often reside in rural areas or maintain closer-knit family and community networks characterized by stronger reciprocal support obligations (103). These dense social networks may provide more robust emotional validation and practical assistance following TBI, thereby bolstering both self-compassion and self-worth. Although we controlled for perceived social support in our regression model, unmeasured qualitative aspects of support—such as cultural appropriateness, integration into daily life, and alignment with self-worth sources—may differ systematically across education levels.

### 4.4 Practical implications

The findings of this study provide important practical guidance for clinical interventions in patients with TBI by revealing the heterogeneous profiles of the relationship between self-compassion and self-worth and their influencing factors. This underscores the necessity of adopting a person-centered approach to develop precise psychological rehabilitation strategies. Specifically, the identified three latent profiles can serve as a foundation for clinical screening, assisting medical teams in quickly categorizing patients and matching targeted interventions, thereby optimizing resource allocation and enhancing treatment efficacy.

#### 4.4.1 Profile-specific intervention recommendations

Class 1: Moderate Self-Compassion – Low Self-Worth Group. This profile represents the highest-risk subgroup, characterized by a disconnect where self-compassion fails to translate into stable self-worth. For these patients, we recommend a multi-component intervention approach. First, Compassion-Focused Therapy (CFT) should be prioritized, specifically targeting the self-worth reconstruction pathway. CFT’s emphasis on developing the “soothing system” through compassionate imagery and self-reassurance exercises can address the self-criticism that may underlie low self-worth despite moderate self-compassion. Prior research by Ownsworth and Haslam (23) demonstrated that integrating CFT with cognitive retraining produced significant improvements in self-evaluation among brain injury patients. Second, Cognitive-Behavioral Therapy (CBT) modules focusing on contingent self-worth restructuring should be incorporated. Given that this profile shows vulnerability in self-worth contingencies, therapeutic work should help patients identify and challenge maladaptive self-worth bases (e.g., appearance, occupational achievement) that have been disrupted by injury, while cultivating intrinsic sources of self-worth (e.g., personal values, relationships). Third, social support enhancement interventions are critical, as this group demonstrated the lowest social support levels (*M* = 2.164). Concrete approaches include family psychoeducation sessions to improve caregivers’ capacity for emotional validation, peer support group participation connecting patients with successfully rehabilitated individuals, and community resource linkage to address instrumental support gaps.

Class 2: High Self-Compassion – High Self-Worth Group. This profile represents patients with optimal psychological adaptation who require maintenance-focused rather than intensive interventions. Recommended approaches include preventive mindfulness-based relapse prevention to sustain high self-compassion levels, regular psychological monitoring (quarterly assessments) to detect early signs of decline, and resilience-building psychoeducation empowering patients as peer mentors for lower-profile patients. These patients may also serve as valuable resources in group interventions, modeling adaptive self-perception strategies for other participants.

Class 3: Moderate Self-Compassion – Moderate Self-Worth Group. As the largest subgroup with balanced but potentially fragile self-perception, these patients benefit from standard supportive interventions with targeted enhancement: Brief self-compassion training using Neff’s Mindful Self-Compassion (MSC) protocol adapted for TBI populations, focusing on the self-kindness and common humanity components; psychoeducation addressing post-traumatic identity adjustment and normalizing psychological challenges following TBI; and booster sessions during high-risk periods (e.g., rehabilitation setbacks, anniversary reactions) to prevent deterioration into lower profiles.

#### 4.4.2 Practice implications of social support and death anxiety

The discovery of social support as a key protective factor suggests that clinicians should incorporate social network reconstruction into rehabilitation plans. For instance, providing emotional and instrumental resources through family involvement groups or community support programs can significantly enhance patients’ self-acceptance and buffer against self-worth crises, consistent with empirical evidence of social support in brain injury recovery. In practical terms, mobile application-based virtual support networks could be developed to provide remote interventions for patients in urban–rural disparity, overcoming geographical barriers and enhancing accessibility. Simultaneously, the negative predictive role of death anxiety emphasizes the need to integrate existential or meaning-oriented therapies, such as managing death anxiety modules, to help patients reconstruct life narratives and reduce existential threats, thereby indirectly enhancing self-worth. This strategy is particularly suitable for the high-anxiety subgroup and can be combined with self-compassion induction exercises to reduce the activation of the threat response system and improve overall psychological adaptation. Given the significant predictive role of death anxiety, particularly for Class 2 membership, we recommend integrating existential therapy components across all profiles. Meaning-centered interventions, adapted from Breitbart’s work with cancer patients, can help TBI patients reconstruct life narratives incorporating the trauma experience. For patients with elevated death anxiety, specific modules addressing mortality acceptance—such as life review exercises and values clarification—can reduce existential distress and indirectly support self-worth stability.

#### 4.4.3 Gender-responsive intervention design

The finding that women were more likely to belong to the high self-compassion–high self-worth profile, while men were overrepresented in lower profiles, has important implications for gender-responsive care. Interventions for male TBI patients should explicitly address barriers to self-compassion, including rigid masculinity norms that discourage emotional vulnerability and self-kindness. Group interventions segregated by gender may facilitate more open discussion of these barriers, and psychoeducation normalizing self-compassion as a strength rather than weakness may improve engagement.

The independent effects of gender and education level further highlight the need for personalized interventions. For male patients, modules should be designed to challenge rigid self-norms and promote self-compassion expression to reduce the risk of falling into low self-worth profiles; for those with lower education, simplified, visually aided intervention forms can leverage their internal resilience advantages to strengthen positive self-perception. On a broader scale, this study supports the development of clinical assessment tools based on LPA, such as a digital screening system integrating self-compassion and self-worth scales for early identification of at-risk subtypes and guiding multidisciplinary teams (e.g., neurosurgeons, psychotherapists, and social workers) in collaborative interventions. Meta-analytic evidence indicates that self-compassion interventions overall can improve psychosocial outcomes, but the unique neurobiological foundations of brain injury must be considered to avoid limited efficacy in severe cases, such as compassion imagery interventions. Additionally, at the policy level, these findings could drive updates in public health guidelines, such as incorporating self-compassion training modules into TBI rehabilitation protocols in China’s tiered healthcare system, to enhance patient quality of life and reduce healthcare burdens.

#### 4.4.4 Implementation considerations

At the systems level, these findings support the development of a stepped-care model for TBI psychological rehabilitation. Initial screening using abbreviated self-compassion and self-worth measures could efficiently stratify patients into risk categories, with intervention intensity matched to profile membership. A digital screening tool integrating these assessments could be embedded in electronic health records, prompting automated referrals to appropriate intervention levels.

For resource-constrained settings, particularly in low- and middle-income countries where TBI prevalence is highest, scalable interventions such as mobile application–delivered self-compassion exercises or trained lay counselor–facilitated support groups offer pragmatic alternatives to specialist-delivered therapy. The social support finding specifically supports investment in community health worker programs to extend psychological support reach.

### 4.5 Limitations and future research directions

Despite providing empirical evidence for the heterogeneity of the relationship between self-compassion and self-worth in patients with TBI, this study has several limitations that require cautious interpretation of the results and guidance for future research. First, the cross-sectional design captures variable associations at a single time point, preventing the establishment of causal relationships. For example, the positive correlation between social support and the High self-compassion - high self-worth profile may stem from bidirectional influences rather than a unidirectional protective effect, which aligns with common challenges in psychological research on chronic diseases. Future longitudinal studies could employ multi-wave tracking designs to examine the dynamic evolution of these variables, clarifying temporal sequences and potential mediating pathways.

Second, regarding latent profile analysis methodology, although sensitivity analyses supported the robustness of our three-class solution, the smallest class (Class 1: Moderate self-compassion - low self-worth) comprised 18.8% of the sample. While this proportion exceeds established minimum thresholds and demonstrated stability in cross-validation, future studies with larger samples should attempt to replicate this finding to confirm the generalizability of this subgroup. Additionally, LPA results can be sensitive to indicator selection; future research could explore alternative operationalizations of self-compassion and self-worth dimensions to validate the profile structure.

Third, the sample recruitment was limited to four tertiary hospitals in Hebei Province, China, using convenience sampling, which may introduce selection bias and limit the generalizability of the results. Given that the global prevalence of TBI is higher in low- and middle-income countries, and cultural factors may moderate self-compassion expression, the geographic and cultural homogeneity of this sample may underestimate cross-cultural heterogeneity. Additionally, the higher proportion of males in the sample, predominantly urban residents, may amplify the effects of gender and urban–rural differences while neglecting the unique psychological needs of rural or minority populations.

Fourth, core variables relied on self-reported measures, which may be influenced by social desirability bias or recall bias, especially among patients with impaired cognitive function post-TBI, whose self-awareness may be limited, leading to measurement errors. Although this study ruled out severe common method bias through Harman’s single-factor test, future studies could integrate multi-method assessments, such as incorporating family reports or objective behavioral indicators, to enhance measurement validity. Fifth, potential confounding factors were not adequately controlled, such as comorbid neurological disorders, medication treatment, or quantification of trauma severity, which may mediate the association between self-compassion and self-worth. We examined death anxiety as a targeted existential construct; future studies should incorporate broader anxiety phenotypes (e.g., generalized anxiety, post-traumatic stress symptoms, fear avoidance) to determine whether different anxiety domains show distinct associations with self-compassion–self-worth profiles.

To address these limitations, future research could expand in the following directions. First, conducting longitudinal or prospective cohort studies to track the profile evolution of TBI patients from the acute to chronic phases and integrating growth mixture models to capture trajectory heterogeneity would help clarify timing and lasting effects of interventions. Second, employing multi-center, multi-cultural sample designs, such as international collaborative studies, could validate the universality and cultural specificity of profile patterns and explore the moderating effects of socioeconomic status. Third, incorporating neurobiological indicators, such as functional magnetic resonance imaging to assess prefrontal cortex activation or biomarkers (e.g., cortisol levels) as covariates, would bridge the gap between psychological heterogeneity and neurobiological mechanisms, deepening the understanding of the neurobiological basis of self-compassion. Fourth, conducting randomized controlled trials to test profile-based personalized interventions, such as comparing the efficacy of compassion-focused therapy across different subgroups and assessing cost-effectiveness to guide clinical guideline formulation. Finally, exploring advanced statistical models, such as structural equation modeling or network analysis, to examine the mediating/moderating mechanisms of death anxiety and social support, and integrating machine learning algorithms to predict profile membership risks, could lead to the development of digital screening tools, enhancing the precision of preventive interventions. These directions would not only address the shortcomings of this study but also advance the accumulation of evidence in the field of psychological rehabilitation for TBI, ultimately improving patients’ quality of life.

## 5 Conclusion

This study revealed significant heterogeneity in the relationship between self-compassion and self-worth in patients with TBI through LPA, identifying three latent subtypes and confirming social support, death anxiety, gender, and education level as key influencing factors. These findings expand the existing literature on post-traumatic psychological heterogeneity, emphasizing the importance of examining self-perception from a person-centered perspective and providing an empirical basis for precise interventions. Specifically, by enhancing social support and alleviating death anxiety, it is possible to effectively improve patients’ levels of self-compassion, thereby buffering against the crisis of self-worth triggered by trauma and promoting overall quality of life improvement. Future research should further validate the longitudinal dynamics of these mechanisms and develop integrated models that incorporate neurocognitive indicators to promote evidence-based practices in TBI rehabilitation, meeting the needs of global patient populations.

## Data Availability

The raw data supporting the conclusions of this article will be made available by the authors, without undue reservation.
